# GBS as unusual presentation of neurobrucellosis: A rare case report

**DOI:** 10.1016/j.radcr.2024.02.067

**Published:** 2024-03-10

**Authors:** Ghazaleh Jamalipour Soufi, Ali Hekmatnia, Farzaneh Hekmatnia, Andrew Parviz Zarei, Shamim Shafieyoon, Sara Azizollahi, Farshad Riahi

**Affiliations:** a Department of Radiology, Isfahan University of Medical Sciences, Isfahan, Iran; b Department of Radiology, St George's Hospital, London, UK; c Department of Medicine, The Princes Alexandra Hospital, London, UK

**Keywords:** Brucellosis, Guillain-Barré syndrome, Neurobrucellosis

## Abstract

Brucellosis is a zoonotic disease caused by small intracellular aerobic Gram-negative bacilli**.** The literature has frequently documented instances of the gastrointestinal, hepatobiliary, and skeletal systems being involved. In 3%-5% of brucellosis patients, neurobrucellosis has been identified. Guillain-Barré syndrome (GBS) is a disorder of the peripheral nervous system. Acute peripheral neuropathy mimicking GBS caused by brucellosis is rarely reported. Our case is of a 34-year-old male presenting with a 3-week history of weakness in the upper limbs. There was a clear history of milk product consumption preceding the onset of symptoms. Examination showed paraesthesia and muscles paralysis. Brucellosis was confirmed via blood test, and GBS was confirmed via imaging and neuroelectrophysiological assessment. The patient was treated with plasma exchange (PLEX) and commenced on rifampicin, doxycycline and gentamicin during their hospitalization. The patient was discharged with a course of rifampicin and doxycycline to complete. In patients with acute paralysis and GBS-like symptoms, Imaging should be done in addition to serological tests for brucellosis.

## Introduction

Brucellosis is a zoonotic disease caused by the bacteria Brucella, a small Gram-negative coccobacillus. Brucella functions as a facultative intracellular parasite causing chronic disease. The disease can be transmitted from animals to humans through direct contact or through the consumption of unpasteurized milk derivatives [1]. It is a significant public health concern in numerous nations worldwide where it is endemic, including developing countries like Turkey. The literature has frequently documented instances of the gastrointestinal, hepatobillary, and skeletal systems being involved. Neurologic abnormalities have been found in 3–5% of brucellosis patients. Muscle rigidity, fever, and headaches are the most frequent clinical presentation of neurobrucellosis. Motor, sensory and mental disorders may also manifest [2]. For the diagnosis of neurobrucellosis, direct or indirect evidence may consist of brucellosis in blood culture and the identification of specific antibodies in cerebrospinal fluid (CSF) [3,4].

Guillain-Barré syndrome (GBS) is a disorder of the peripheral nervous system characterized by a rapidly progressing, ascending, flaccid paralysis with decreased or absent reflexes [5]. Segmental demyelination of sensory and motor neurons in peripheral nerves and various nerve roots are the main lesions seen; nonetheless, brucella-induced GBS is extremely uncommon. The literature presents diverging opinions regarding the diagnostic criteria for neurobrucellosis. Some authors propose that the diagnosis should be established on the basis of clinical neurological symptoms, while others advocate for microbiological and biochemical evidence derived from CSF [6].

Currently, we present a 34-year-old male patient with active brucellosis along with GBS that has been diagnosed both clinically and with laboratory result.

## Case presentation

A 34-year-old male was referred to the Ayatollah Kashani Hospital of Isfahan University of Medical Sciences presenting with upper and lower limb weakness. He described a 3-week history of progressively worsening paresis and paresthesia in the upper and lower limbs. At the time of referral symptoms were worse in the upper than lower limbs. He denied any fever or night sweats and there were no gastrointestinal or respiratory symptoms. He gave a clear history of the consumption of traditional ice cream prior to the onset of his symptoms.

Past medical history includes anxiety, depression and hypertension. His drug history includes fluoxetine, valsartan, gabapentin and sodium valproate.

Neurological examination was carried out on the patient. The cranial nerves were intact and normal on examination. Power was assessed according to the Medical Research Council (MRC) muscle power scale. There was a power of 3/5 bilaterally in both distal and proximal muscles of the upper limb. In the lower limbs power was 4/5 bilaterally in both proximal and distal muscles. On assessment of reflexes, there was reduced brachioradialis reflexes bilaterally. Babinski sign was positive bilaterally. He had impaired proprioception in his lower extremities, with otherwise normal sensory assessment.

Laboratory analysis of blood and CSF was performed. Results showed a raised creatinine phosphokinase (CPK). The complete blood count (CBC), C-reactive protein (CRP), erythrocyte sedimentation rate (ESR), renal, and liver function, and blood sugar (BS) were normal (Table 1). Also, the Wright (1/640), Coombs Wright (1/1280), and 2-Mercaptoethanol (2-ME) (1/320) tests were positive for active brucellosis. A lumbar puncture (LP) performed on the third day of admission showed a classic albumin–cytological dissociation (white blood cell counts of 5 cells/cubic millimeter, protein level of 220 mg/dL [normal value: 15-60 mg/dL], and a CSF glucose level of 100 mg/dL [normal value: 50-80 mg/ dl]). Wright and 2-Mercaptoethanol (2-ME) testing and culture of CSF were negative.

**Table 1:** Laboratory finding of our case.

Test	Result	Normal	Test	Result	Normal
WBC (/m^3^)	7100	4000-11000	AST (U/L)	25.2	1-37
Neutrophils (%)	70.4	50-70	ESR (1 h) (mm/h)	3	0-10
Lymphocyte (%)	25.6	20-40	BS (mg/dL)	108	70-100
RBC (10^12^/L)	5.27	4.5-5.9	CPK (U/L)	232	1-171
Hb (g/dL)	15.2	14-17.5	Cr (mmol/L)	1.1	0.7-1.3
MCV (fl)	80.3	80-96	BUN	14	8-25
PLT (10^3^ /μL)	237	150-450	K (mmol/L)	3.5	3.8-5
CRP (mg/dL)	0/6	<5	Na (mmol/L)	136	136-145
ALT (U/L)	30.5	1-41	Mg (mmol/L)	2.5	1.8-2.6

WBC, white blood cell; HB, hemoglobin; PLT, platelets; RBC, red blood cell; CRP, C-reactive protein; MCV, mean corpuscular volume; CPK, creatine phosphokinase; Cr, Creatinine; BUN, blood urea nitrogen; AST, aspartate aminotransferase; ESR, erythrocyte sedimentation rate; ALT, alanine aminotransferase.

During electromyography and nerve conduction velocity (EMG-NCV), it was discovered that there was evidence of axonal and demyelinating polyneuropathy, indicative of the subacute manifestation of GBS. In the lower extremities, the rate of sensory nerve transmission was diminished. Pyramidal and meningeal irritation signs were both negative.

**Fig. 1:**
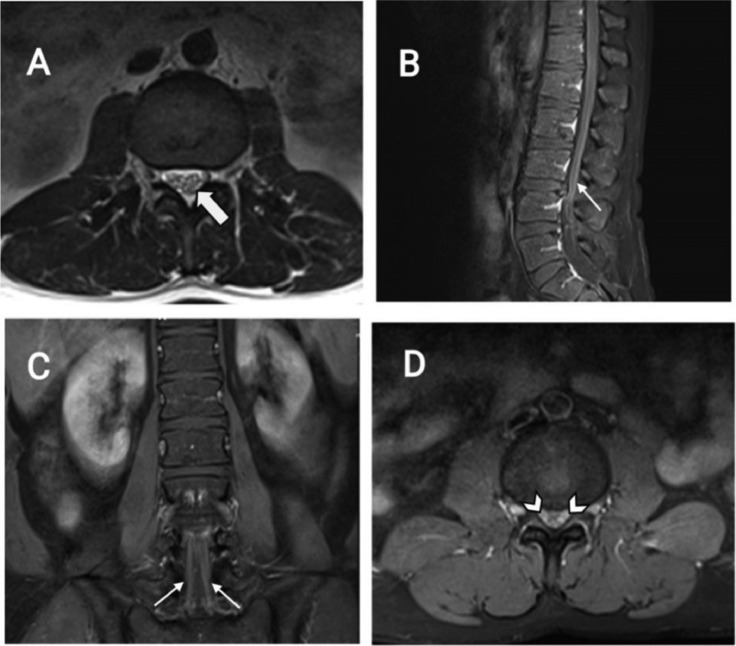
(A) T2 –Weighted Axial image demonstrates Cauda equina nerve root thickening(large arrow); (B) T1-Sagittal Fat-suppressed post contrast image and (C) T1-Coronal Fat-suppressed post contrast image shows diffuse Cauda equina superficial enhancement (arrows); (D) T1-Axial Fat-suppressed post contrast image also shows diffuse Cauda equina superficial enhancement with meningeal enhancement additionally (arrowheads).

Imaging revealed no signs of infarction or haemorrhage on a computed tomography (CT) scan of the brain. The thoracolumbar magnetic resonance imaging (MRI) demonstrated a central disc protrusion and canal and foraminal narrowing at L3-L4 and L4-L5 levels. The MRI also demonstrated diffuse cauda equina thickening and enhancement associated with diffuse meningeal enhancement with upward extension to the lower thoracic level, suggesting GBS (Fig. 1). A diagnosis of active brucellosis, accompanied by a presentation resembling GBS, was subsequently made for the patient.

As a result of the limited accessibility and high cost of intravenous immune globulin (IVIG), plasma exchange (PLEX) was chosen. The patient underwent a total of twelve sessions of this. Furthermore, he was prescribed a regimen of antibiotics to treat the brucellosis: gentamicin 80 mg (daily), doxycycline 100 mg (BID), and rifampin 300 mg (QID). The muscle power in the lower extremities continued to improve with the absence of tendon reflexes after 8 days of treatment. The patient was discharged after 15 days with instructions to continue the therapies. For discharge, rifampicin 600 mg/day and doxycycline 200 mg/day were prescribed for the patient.

The progress of our patient was monitored and at a 4-week follow-up they demonstrated a partial recovery with the ability to mobilize independently.

## Discussion

Neurobrucellosis was first documented by Hughes in 1896. Although neurobrucellosis is uncommon worldwide, it is reported to be very common in brucellosis-endemic nations. It is believed that neurobrucellosis accounts for 0.5% of all instances of community-acquired central nervous system (CNS) infections [7].

The etiology of the acute phase of polyradiculopathy caused by *Brucella* infection, which induces an immune response against autoantigens, remains unknown. It could be the result of inflammatory and immunological reactions. A study on animals showed that acute paralysis and GBS symptoms were brought on by autoantibodies against myelin gangliosides, which were expressed on the surface of *Brucella* [8]. General symptoms that may be experienced by individuals diagnosed with neurobrucellosis include fever, myalgia, perspiration, lower back pain, fatigue, appetite loss, arthralgia, and vomiting. The most prominent neurological manifestation observed in the majority of patients with neurobrucellosis is a severe headache. Additionally, muscle weakness, variations in DTRs, agitation, apathy, disorientation, ataxia, neck rigidity, diplopia, blurred vision, and behavioural disorders may manifest clinically in patients [9].

In most cases, neurobrucellosis is identified through a history of milk or dairy product consumption meeting any of the subsequent criteria [10]. (1) the absence of symptoms resembling alternative neurological disorders; [2] the detection of anti-Brucella antibodies or the isolation of Brucella species from cerebrospinal fluid (CSF); [3] abnormally high protein and glucose levels in CSF; [4] the diagnostic support offered by a cranial magnetic resonance imaging (MRI) or computed tomography (CT) scan; and [5] the reduction of symptoms following appropriate treatment, as indicated by a reduction in the lymphocyte and protein counts in CSF.

CSF lymphocytic pleocytosis is present in 91% of Brucella meningitis cases, along with elevated protein levels and normal or low glucose. When neurobrucellosis impacts the cerebellum, the CSF contains an increased amount of protein but no leukocytosis. A positive CSF culture is often observed in patients with neurobrucellosis, whereas a positive blood culture was detected in only 30% of cases and 14% of patients with neurobrucellosis [11].

In our investigation, we present a 34-year-old male who exhibited weakness in his upper limbs and a medical history of brucellosis due to the consumption of contaminated milk products. He showed no characteristic symptoms of any other known neurological disorder and tested positive on a serological test (Wright reaction: 1/640). Even though the antibody titers could not be performed, this test result was crucial in inducing suspicion of brucellosis. The diagnosis of GBS was confirmed according to the following characteristics: absent DTRs in the lower extremities and reduced brachioradialis reflexes bilaterally in the upper limb; albuminocytologic dissociation shown in CSF analysis; and EMG-NCV revealed evidence of axonal and demyelinating polyneuropathy.

In 2022, Li et al. [12] reported a 55-year-old male referred with a presentation of fatigue, intermittent fever, and waist pain for over 3 months. A blood test revealed that the patient had Brucellosis. Although CSF analysis demonstrated the expected changes in protein-cell separation, all pathogen-related tests produced negative results. Furthermore, anti-GM1 antibody IgG and IgM, as well as anti-GD1b antibody IgG and IgM, were found in all blood and CSF samples. In addition, the EMG-NCV showed an increase in limb F wave delay. This investigation is consistent with our findings, although we did not have access to the antibody titration data.

In 2021, Alanazi et al. [6] reported 19 cases of brucellosis with GBS-like symptoms, with a range of 9-62 years. A fever was present in 8 patients, whereas 36.8% exhibited no symptoms. In nine of the fourteen patients, albuminocytological dissociation was observed. In 8 patients, EMG-NCV confirmed demyelination polyneuropathy; in 6 patients, axonal polyneuropathy; and in 1 patient, a combination of axona,l and demyelinating polyneuropathy. Additionally, 3 patients exhibited root enhancement on MRI of the spine. In 2022, Daoud et al. [13] presented a 54-year-old woman, with no past medical history that was diagnosed with brucellosis with a GBS-like presentation. The patient was referred with a presentation of bilateral facial muscle paralysis and a rapidly progressive, ascending, and symmetrical weakness. Brucellosis was confirmed by an increased protein level (0.7 g/L) in CSF and raised Coombs Wright titration (1/160). The above studies were in line with our study.

Although imaging is not used frequently to diagnose GBS, MRI images show thickening and significant enhancement of the anterior spinal nerve roots, particularly in the cauda and conus medullaris regions [14]. In our case MRI also demonstrated diffuse cauda equina thickening and enhancement associated with diffuse meningeal enhancement with upward extension to the lower thoracic level. In 2014, Elzein et al. [15] presented a 54-year-old man with a diagnosis of GBS secondary to active brucellosis. In the MRI imaging, subtle edema in the right parietal lobe with associated leptomeningeal enhancement was seen. In contrast to our study, Doya et al. [16] reported a 4-year-old boy with a GBS-related brucellosis infection without any remarkable evidence of GBS on a whole brain and spinal MRI. In this case, the confirmation of a GBS diagnosis was only by CSF and electrophysiological examination. In similar research, Li et al. [12] described a 55-year-old male with brucellosis-induced GBS. Furthermore, there were no visible abnormalities in this patient's whole spine MRI or head CT scan.

Although both IVIG and PLEX are first-line treatment options for GBS, physicians may attempt a second round of sustained treatment if the first round of PLEX in patients with severe GBS fails to produce an improvement following the conclusion of IVIG therapy. Nevertheless, the available evidence is insufficient to substantiate the superiority of combination therapy [17]. Therefore, due to lack of availability of IVIG and its high cost, the patient received PLEX for 12 sessions and IVIG was not administered. In a study conducted by Alanazi et al. [6], 3 patients (15.8%) were administered IVIG alone, 7 patients (36.8%) were administered PLEX alone, three patients (15.8%) were administered both IVIG and PLEX, and 6 patients (31.6%) were not administered either IVIG or PLEX. Antibiotics were given to all of the patients. One patient (5.3%) died, while 16 patients (84.2%) regained their ability to walk. Two weeks to 1 year was the range for walking recuperation. Elzein et al. [15] and Babamahmoodi et al. [18] both documented 2 cases of GBS associated with brucellosis infection, which manifested as paralysis of the eyes and face, numbness in the lower and upper extremities, and muscle weakness. In contrast to our study, both patients received suitable IVIG therapy and attained favorable outcomes.

## Conclusion

In conclusion, when a patient arrives with neurological symptoms, including paraparesis, and has positive serological testing for brucellosis, one of the diagnoses to investigate is GBS involvement of the CNS, and accordingly, a spine MRI is required.

## Patient consent

Complete written informed consent was obtained from the patient for the publication of this study and accompanying images.
